# Distinguishing between help and harm: Helper T cell subsets and immune-related adverse events

**DOI:** 10.1172/JCI184310

**Published:** 2024-10-15

**Authors:** Alexandra M. Haugh, Adil I. Daud

**Affiliations:** 1 Department of Medicine, Massachusetts General Hospital, Boston, Massachusetts, USA.; 2 Department of Medicine, Division of Hematology/Oncology, UCSF, San Francisco, California, USA.

## Abstract

The precise conditions by which cytokines drive cancer is relevant to improving immune checkpoint inhibition (ICI) responses while decreasing toxicity. In this issue of the *JCI*, Kao et al. investigated T helper cell pathways in patients with solid tumors receiving ICI. The authors evaluated T cell populations, cytokine signatures, immune related adverse events (irAEs), and survival outcomes. Patients with a history of autoimmune disorders were more likely to develop irAEs. Notably, blood samples from patients on treatment showed that elevations in IL-5, IL-6, IL-17f, and TNF-α were associated with an increased risk for grade 2 or higher irAEs. Moreover, IL-6 was associated with decreased objective response rate and worse cancer-specific and all-cause mortality. These findings may help guide decisions for optimizing ICI efficacy while minimizing toxicity and suggest that IL-6 blockade may improve response and decrease toxicity in solid tumors.

## Antitumor and autoimmune responses

The interaction between CD4^+^ helper T cell subsets and the development of antitumor adaptive immunity and autoimmunity is a topic of active investigation. While CD8^+^ killer T cells are crucial players in the adaptive immune response to immune checkpoint inhibition (ICI), it is increasingly appreciated that CD4^+^ helper T cells polarize, assist, and interact with dendritic T cells as well as other innate immune cells to drive both antitumor and autoreactive immunity observed with ICI (1–3). Various cytokines have been implicated in driving polarization of helper T cells into at least seven subsets depending on the immune stimulus encountered.

Three of these T helper cell pathways, which have well-established implications in antitumor immune responses and the development of autoimmune diseases, were the primary subject of investigation in this issue of the *JCI* by Kao et al. (4). The Th1 pathway, driven by the cytokines IL-12 and IFN-γ, is thought to play a key role in response to intracellular pathogens and is thus implicated in antiviral immunity and in adaptive immune rejection of tumors (5, 6). Th2 immunity, driven by IL-4, IL-5, and IL-13 is thought to mediate antiparasite immunity and also plays a role in many allergic disorders such as asthma (7). The Th17 pathway, mediated primarily by IL-1b, IL-17, and IL-21/IL-23, plays a role in barrier immunity in the gut and skin and is associated with a spectrum of autoimmune disorders including psoriasis, rheumatoid arthritis, and inflammatory bowel disease (8–10). Increasing evidence has also demonstrated a role for Th17 immunity in antitumor immune responses (1, 11, 12).

In this study, Kao et al. (4) report on the prospective enrollment of 111 patients with cancer who were undergoing standard of care treatment with ICI involving serial blood sampling and clinical follow up. Patients with a range of primary tumor types were enrolled, including those with common cancers, including gastrointestinal (GI) (34.2%), genitourinary (GU) (30.6%), and skin cancers (12.6%). Grade 2 or greater immune related adverse events (irAEs), the primary outcome assessed in this study, occurred in 45 of the 111 patients enrolled (40.5%) with an average of 7.4 months of on-treatment followup. A majority (*n* = 33 of 45, 73.3%) of irAE occurred within the first six months of ICI initiation but there were some identified at later time points (*n* = 12 of 45, 26.7%).

Kao, Charmsaz, and colleagues (4) notably included populations underrepresented in immunotherapy research, including 31 patients who identified as Black (27.9%) and 14 (12.6%) with a history of preexisting autoimmune disease. While both of these populations represent a substantial proportion of real-world patients treated with immunotherapy, they generally have either been excluded from or underrepresented in clinical trials and associated translational research efforts. Thus, there is a gap in evidence-based guidance and understanding of how to treat these patient populations and efforts to include them are warranted.

## Cytokines that drive immune related adverse events

Blood samples collected in Kao et al. (4) were analyzed to assess for the predictive and prognostic importance of cytokine profile and circulating immune cells at three time points: (a) baseline pretreatment (*n* = 111); (b) on treatment prior to the development of irAEs (*n* = 102), and (c) at the time of irAE development (*n* = 24). Importantly, peripheral blood samples from patients who developed irAE had to be collected prior to any immunosuppressive therapy, making this data point logistically difficult and limiting the sample size.

None of the 32 cytokines assessed in baseline blood samples were independently predictive of the development of irAEs. There were notable differences in baseline cytokine levels based on primary tumor histology, particularly involving IL-8, IL-17f, IP-10, and RANTES. Given the this heterogeneity and the lack of predictive value of any individual cytokine, the authors evaluated circulating immune cell populations. cytometry by time-of-flight (CyTOF) was performed using a 37-antibody panel that included markers of Th cell subset (type-1, type-2, type-17), regulatory T cells (FOXP3), naive T cells states, and T cell activation and exhaustion markers such as PD1, 41BB, and LAG3. While baseline cytokine profiles were not predictive of irAE, patients who developed irAEs had more circulating Th17 cells at baseline compared with those who were unaffected by irAEs.

Baseline cytokine levels and circulating immune cells were then compared to paired samples collected while patients were on therapy. The median time on ICI prior to sample collection compared with the time of irAE onset suggested that in many instances pretreatment specimens were drawn relatively close to the point at which irAE clinical symptoms became apparent. Plasma cytokine signatures were classified as type 2 (including IL-5, IL-13, and IL-25), Th17 related (IL-6 and IL-17f), and type 1 (TNF-α). Of these six cytokines, IL-5, IL-6, IL-17f, and TNF-α individually stratified patients for higher risk of irAE. Further, when stratified based on the organ involved by toxicity, IL-5, IL-6, and TNF- α were differentially elevated. TNF-α abundance was more common in pulmonary toxicity, while IL-6 elevation was commonly associated with enterocolitis. In regards to on-treatment circulating immune populations, increased Th2EM and/or Th17 cell subsets following initiation of ICI correlated with irAEs.

Cytokine profiles were finally compared between the small patient cohort with plasma collected after irAE onset prior to any systemic immunosuppression and a patient cohort with matched time points in the absence of irAEs. This analysis found only IL-6 and IL-17f to be differentially expressed amongst patients with irAEs, further underscoring the importance of Th17-related cytokines in driving irAEs.

## Clinical implications

**Figure 1:**
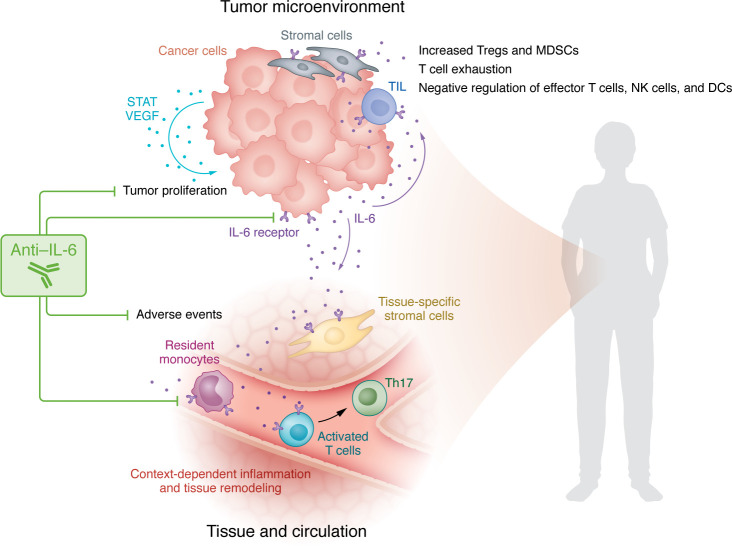
IL-6 blockade may potentiate antitumor immune responses to ICI in addition to abrogating toxicity risk. Kao et al. (4) showed that patients with solid tumors receiving ICI had elevations in cytokines, including IL-6, and were at risk for irAEs. Importantly, IL-6 correlated with decreased ORR and worse mortality (4). In the tumor microenvironment (TME), IL-6 increases the quantity of Tregs and MDSCs, promotes T cell exhaustion, negatively regulates effector T cells, and modulates NK cells and dendritic cells. IL-6 also increases the production of STAT and VEGF in tumor cells with tumor proliferating effects. Systemically, IL-6 promotes differentiation of activated T cells and increases inflammation and tissue remodeling via tissue-resident monocytes and stromal cells. Notably, tumor cells and cells within the tumor microenvironment, including tumor-infiltrating lymphocytes and stromal cells, express IL-6 receptors. Tissue and circulating immune cells involved with irAEs also express IL-6 receptors. Targeting the IL-6 receptor with blocking antibodies in solid tumors treated with ICI may improve the response to treatment while decreasing the risk for irAEs.

Of the cytokines investigated, IL-6 was notably the only cytokine associated with decreased objective response rate (ORR) and worse cancer-specific and all-cause mortality. This observation is supported by preclinical data suggesting that IL-6 blockade may potentiate antitumor immune responses in addition to abrogating risk for toxicity (Figure 1) (13, 14). A recently presented phase 2 trial enrolled patients with unresectable melanoma to receive an anti-IL-6 antibody (tocilizumab) in addition to flipped dose ICIs (ipilimumab and nivolumab) and showed a best overall response rate of 57% and grade 3/4 toxicity rate of 22% (15). Another trial enrolled patients with melanoma to receive tocilizumab in addition to standard dose ipilimumab and nivolumab and found evidence of increased Th17-associated gene expression with biweekly dosing of tocilizumab; a small cohort of patients was thus treated with dose-dense weekly tocilizumab with findings suggestive of improved ORR and somewhat decreased toxicity (16).

Investigation into the role of cytokines in cancer is often complicated by the fact that their role seems in many cases to be tumor and context dependent; the correlation between IL-6 and outcomes in the spectrum of malignancies represented in Kao et al. (4) is, therefore, particularly notable and suggests that IL-6 blockade may be relevant to improving responses and decreasing toxicity in other solid tumors treated with ICI.

The role of IL-17 in promoting versus hindering tumor cell growth and antitumor immune responses is controversial with several prior studies demonstrating mixed results (17–20). A higher baseline IL-17 associated gene expression signature in pretreatment tumor specimens was recently shown to correlate with improved overall survival and longer progression free survival with dual ICI in patients with melanoma (12). Higher baseline plasma IL-17A levels also correlated with response to dual ICI but not to single-agent ICI (12). Adoptive transfer of Th17-polarized CD4^+^ T cells has additionally been shown to generate particularly potent antitumor responses in preclinical models (21, 22). While incompletely understood, this response is thought to be due, at least in part, to an ability to resist senescence and apoptosis as well as stem memory potential that allows Th17-polarized cells to convert to different Th cell phenotypes following transfer (23). Although IL-17 itself was not independently associated with outcomes in Kao,et al. (4), the Th17 pathway, including IL-6 and other signaling components, had important implications for both response to therapy and risk of irAE development. Baseline IL-17f was also one of the 32 cytokines differentially elevated at baseline depending on primary tumor histology, suggesting that the relationship between IL-17 and response might be tumor specific.

A final interesting aspect of this study is the creation of a cytokine score based on the six cytokines found to be independently predictive of the development of irAE. The risk of irAE increased with the number of cytokines elevated, with risk found to be highest when at least four of these six cytokines were elevated in on-treatment samples, a finding that may warrant further investigation in larger scale studies. The clinical relevance of an on-treatment biomarker is likely less meaningful than one that could identify pretreatment patients most at risk for complications, particularly given the long half life of ICI. However, any biomarker that could help guide clinical decision making around balancing toxicity and potential efficacy with ICI could be quite impactful for the large population of patients exposed to these therapies.

## Version 1 10/15/2024

Electronic publication
